# Cardiomiopatía cirrótica – ¿Realidad clínica o simple curiosidad académica? Revisión. Parte 1: definición, epidemiología, anatomía patológica y clínica

**DOI:** 10.31053/1853.0605.v81.n1.44416

**Published:** 2024-03-27

**Authors:** Hugo R Ramos, Mario H Altieri

**Affiliations:** 1 Cátedra de Medicina I-II, Facultad de Ciencias Médicas, Universidad Nacional de Córdoba. División Cardiología, Instituto Modelo de Cardiología Córdoba Argentina; 2 Service de Médecine, Centre Hospitalier Marguerite de Lorraine Mortagne au Perche Francia

**Keywords:** cirrosis hepática, cardiomiopatía, insuficiencia cardíaca, liver cirrhosis, cardiomyopathy, heart failure, cirrose hepática, cardiomiopatía, insuficiéncia cardíaca

## Abstract

La cirrosis avanzada puede provocar alteraciones miocárdicas que constituyen el síndrome de Cardiomiopatía Cirrótica definido como la disfunción cardíaca asociada con cirrosis hepática en ausencia de enfermedad cardíaca preexistente. Su prevalencia es variable de acuerdo a lo reportado por diferentes grupos de investigación debido a que puede mantenerse subclínica o latente hasta que la pone de manifiesto una situación de estrés como una cirugía, hemorragia, infección, trasplante hepático o shunt porto-sistémico intrahepático transyugular. El objetivo de esta revisión es discutir la definición, los fundamentos anátomo-patológicos, fisiopatología, manifestaciones clínicas, criterios de diagnóstico, importancia de los estudios con imágenes, relevancia clínica, tratamiento farmacológico y trasplante hepático.

## Abstract (en)

Severe cirrhosis affecting myocardial function provokes a syndrome called Cirrhotic Cardiomyopathy, defined as cardiac disfunction associated with hepatic cirrhosis in the absence of other known cardiac disease. The prevalence is variable according different groups of investigation owing to the latent or subclinical course until a stressful situation unmask it such as surgery, hemorrhage, infection, hepatic transplant or transjugular intrahepatic porto-systemic shunt. We aimed to review the definition, pathology, pathophysiology, clinical manifestations, diagnostic criteria, images, clinical relevance, pharmacological treatment and hepatic transplantation.

## Resumo

A cirrose avançada pode provocar alterações miocárdicas que são conhecidas como o Síndrome de Cardiomiopatia Cirrótica definida como a disfunção cardíaca associada com cirrose hepática em ausência de doença cardíaca preexistente. A prevalência e variável de acordo com os reportes obtidos por diferentes grupos de investigação devido a que pode permanecer subclínica ou latente até que é manifestada após uma situação de estrese como uma hemorragia, infecção, transplante hepático o shunt porto-sistémico intra-hepático transjugular. O objetivo desta revisão e discutir a definição, os fundamentos anátomo patológicos, fisiopatologia, manifestações clínicas, critérios de diagnostico, importância dos exames com imagens, relevância clínica, tratamento farmacológico e transplante hepático.

**CONCEPTOS CLAVE**

**¿Qué se sabe sobre el tema?**

La cirrosis hepática es grave y progresiva y afecta a otros órganos y sistemas; uno de ellos es el corazón provocando disfunción diastólica y/o sistólica, trastornos del ECG y de la circulación sistémica que no son detectados debido a que la Cardiomiopatía Cirrótica es subclínica en gran parte de su evolución y la mayoría de los pacientes son diagnosticados durante una descompensación.

**¿Qué aporta este trabajo?**

Fundamentos epidemiológicos, patológicos y de la fisiopatología que explican las manifestaciones clínicas y los nuevos criterios de diagnóstico.

**Divulgación**

Cardiomiopatía Cirrótica es la disfunción cardíaca asociada con cirrosis hepática en ausencia de enfermedad cardíaca preexistente. Generalmente pasa desapercibida debido a su evolución subclínica y a que muchos síntomas se superponen con los de cirrosis e hipertensión portal, pero puede manifestarse severamente durante un episodio de descompensación debido a cirugía, infección, sangrado, colocación de TIPS o trasplante hepático. Su diagnóstico oportuno puede prevenir complicaciones graves.

## Introducción

La insuficiencia cardiaca es un síndrome muy prevalente y la carga global de Cardiomiopatía y Miocarditis es de 6.11 millones con 370.000 muertes anuales. Además de la Cardiomiopatía idiopática, las causas son múltiples y la Cardiomiopatía Cirrótica (CMC) es una de ellas, aunque solamente el alcohol es reconocido como agente etiológico de manera que la CMC no se menciona sistemáticamente en los registros de enfermedad cardiovascular. ^1^

El compromiso cardiaco descripto en la cirrosis hepática fue inicialmente atribuido a la toxicidad directa del alcohol y los primeros criterios de diagnóstico de la CMC fueron propuestos en 2005 en Montreal, Canadá, aunque luego de los progresos realizados en las imágenes cardiacas estos fueron actualizados en 2020 por el Cirrhotic Cardiomyopathy Consortium. ^2^ Se ha demostrado que la CMC está involucrada en la ocurrencia del síndrome hépato renal (SHR) y contribuye con una morbimortalidad notable después de una infección, trasplante hepático o posterior a la inserción de un shunt porto-sistémico intrahepático transyugular (Transjugular intrahepatic porto-systemic shunt: TIPS). ^3^ Teniendo en cuenta su carácter poco sintomático fuera de las situaciones de estrés, la prevalencia exacta de esta patología es desconocida.

El objetivo de esta revisión es describir su epidemiología, patogenia, criterios de diagnóstico y relevancia clínica, tratamiento y el impacto en los resultados después del trasplante hepático.

## Materiales y Métodos

Se investigó por MEDLINE (PubMed) entre octubre de 1953 y enero de 2024 con los siguientes términos: "cirrhosis", "cardiomyopathy", "liver cirrosis", "portal hypertension", "heart disease", "portal cirrhosis" and "liver transplantation". Además, se revisaron las referencias de los artículos identificados por esta estrategia y se seleccionaron las fuentes consideradas como las más relevantes para la práctica contemporánea de estudios del mundo real, estudios de cohorte, estudios de corte transversal, ensayos controlados randomizados y estudios de investigación básica. Esta revisión no requirió aprobación del Comité de Ética Institucional y se siguieron los principios de la Declaración de Helsinki.

## Resultados

### Definición

**Figura 1:**
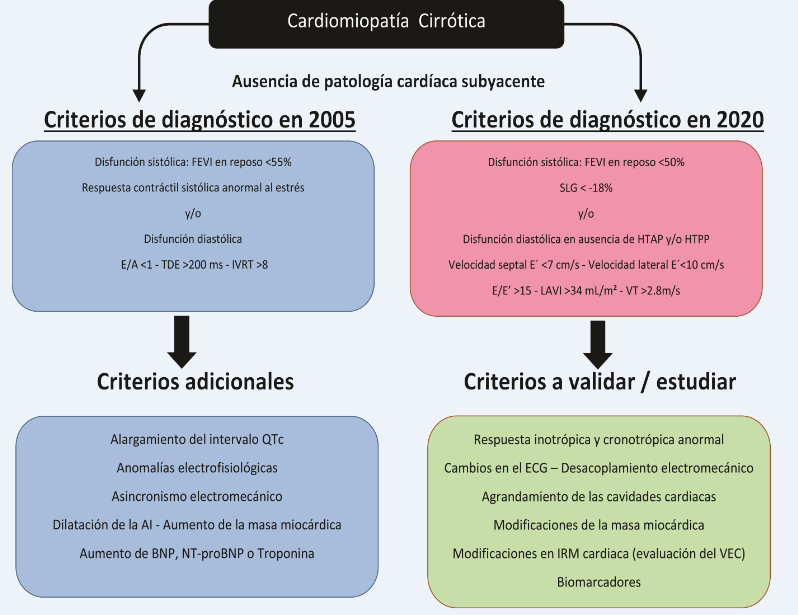
Criterios de diagnóstico de la Cardiomiopatía Cirrótica basados en los Consensos publicados en 2005 y 2020 ^2^ A: velocidad de llenado auricular; AI: aurícula izquierda; BNP: Brain Natriuretic Peptide; E: velocidad pico de llenado diastólico precoz; E': velocidad diastólica precoz anular mitral promedio de los sitios septal y lateral; E/A: relación calculada de E/A; E/E': relación calculada de E/E'; ECG: electrocardiograma; FEVI: fracción de eyección de ventrículo izquierdo; HTAP: hipertensión arterial pulmonar; HTPP: hipertensión porto-pulmonar; IRM: imágenes de resonancia magnética; IVRT: tiempo de relajación isovolumétrico; LAVI: índice de volumen de aurícula izquierda; NT-proBNP: aminoterminal pro-brain natriuretic peptide; QTc: intervalo QT corregido; SLG: strain longitudinal global; VEC: volumen extracelular; TDE: tiempo de desaceleración de onda E; VT: velocidad tricuspídea.

En 1953 Kowalski y Abelmann describieron un síndrome hiperdinámico también llamado hipercinesia circulatoria, en pacientes con cirrosis alcohólica caracterizada por un aumento del volumen minuto (VM), disminución de la resistencia arterial periférica y de la presión arterial. ^4^ Regan y col observaron hace más de cinco décadas la disfunción cardiaca en pacientes con cirrosis alcohólica, ^5^ pero el término de Cardiomiopatia Cirrótica le pertenece a Samuel Lee, acuñado en 1989. ^6^ Conceptualmente, la CMC es la disfunción cardíaca asociada con cirrosis hepática en ausencia de enfermedad cardíaca preexistente. ^2, 6^ Los criterios para el diagnóstico se detallan en la **Figura 1.** Expresa un trastorno circulatorio en el curso de la hipertensión portal (HTP) caracterizada por alteración de la función ventricular diastólica y/o sistólica asociada a anomalías electrofisiológicas. La CMC es independiente de la etiología y la severidad de la hepatopatía, generalmente es asintomática u oligosintomática por mucho tiempo y se la reconoce cuando es expuesta al estrés. ^7-10^

### Epidemiología

La cirrosis hepática es una enfermedad frecuente y provoca 1.16 millones de muertes globalmente, ^11^ y aunque el compromiso cardíaco suele ser un marcador de gravedad, la función cardíaca puede parecer normal o casi normal en reposo hasta que la descompensación la pone en evidencia. Debido a la naturaleza latente de la CMC es difícil establecer con precisión su prevalencia. Chahal y col registraron una prevalencia variable entre 26-81%; ^12^ Shahvaran y col en un metanálisis de 12 estudios siguiendo los criterios de Montreal 2005, encontraron que la prevalencia definida por ecocardiograma 2D (Eco 2D) fue de 61%, mientras que fue 45% cuando se utilizaron criterios de imágenes de doppler tisular (TDI), es decir que las imágenes aisladas de Eco2D podrían sobrediagnosticar CMC. ^13^ Cesari y col usando los criterios de 2020, encontraron una prevalencia con strain longitudinal global (SLG) anormal en 25% de los pacientes y disfunción diastólica avanzada en 10%. ^14^ Razpotnik y col utilizando los criterios 2005 y 2019 encontraron una prevalencia de CMC de alrededor del 60%, siendo la disfunción diastólica más frecuente con los criterios 2005 que con los de 2019 (64.8% vs 7.4%, P <0.0001), mientras la disfunción sistólica fue más frecuente con los criterios 2019 que con los criterios de 2005 (53.3% vs 16.4%, P <0.0001). ^15^ Cuando la respuesta cardíaca anormal fue definida por mediciones con catéter de Swan-Ganz antes de trasplante hepático, Ripoll y col observaron una prevalencia de 10% y aumentó a 25% en el periodo post-reperfusión, sin que estos hallazgos tuvieran correlación con los parámetros de función diastólica en el ecocardiograma. ^16^ El desarrollo de la CMC parece independiente de la etiología de la cirrosis subyacente y la disfunción cardíaca en algunos casos no está asociada con la severidad de la enfermedad hepática y sería independiente de los scores Child-Pugh y MELD. ^17^ Dada esta dificultad para establecer la prevalencia de la CMC, en todo paciente con cirrosis hepática con HTP se deberían buscar las alteraciones cardíacas sugestivas como se describe más abajo.

### Anatomía Patológica

El corazón en ratas con cirrosis experimental tiene significativamente más peso que el de las no cirróticas ^18^ y después de una hemorragia hay abundantes infiltrados de monocitos/macrófagos en el miocardio. ^19^ En humanos con cirrosis de cualquier etiología, los corazones pesan >300 g en 24%-60% de los casos. ^20-22^ En un estudio de 108 casos, Lunseth y col mostraron que en 12 pacientes (11%) hubo hipertrofia y dilatación de ventrículo izquierdo (VI) con dilatación de ventrículo derecho (VD) que no podía ser explicada por otra patología cardíaca concomitante (hipertensión arterial, valvulopatía, enfermedad aterosclerótica u otra). Estos autores describieron lo que podría ser la microscopía típica de la CMC: miocardiocitos edematosos, especialmente en el subendocardio con vacuolización citoplásmica y nuclear acompañado de lipofucsina perinuclear, un pigmento de desgaste debido a la peroxidación de los lípidos de las membranas subcelulares. También observaron fibrosis miocárdica difusa que comprometía solo un segmento de fibra muscular y tejido fibroso entre los espacios en donde las fibras musculares se encontraban rotas de manera transversal; la fragmentación de las fibras musculares fue más frecuente en los corazones de cirróticos que en los corazones hipertróficos no cirróticos. Se observaron infiltrados linfocíticos e histiocíticos sin evidencia de infección o toxinas. Estos hallazgos apoyarían la hipótesis de que la CMC es una entidad específica. ^21^ Ortiz-Olvera y col reportaron 12.7% de hipertrofia de VI en 133 casos de cirrosis alcohólica y no alcohólica, ^20^ mientras que Wehmeyer y col estudiaron 659 cirróticos/19018 autopsias y encontraron una prevalencia de anormalidades cardíacas de 46% para todas las etiologías de cirrosis (cirrosis alcohólica, viral o criptogénica), con la excepción de la esteato-hepatitis no alcohólica (NASH) que tuvo 71% de hipertrofia de VI. Además, 24% de los cirróticos tenía un peso cardíaco ≥500 g, y para todo el grupo el peso del corazón fue significativamente mayor que el de no cirróticos (436 ± 145 g vs 386 ± 82 g; P=0.038). ^22^ La hipertrofia de VD fue detectada en 24% de los pacientes y fue prevalente en todos los grupos, con excepción de la hepatitis viral. La dilatación de aurícula izquierda y de VI y la enfermedad coronaria aterosclerótica estuvieron significativamente más asociadas con NASH que con las otras etiologías, probablemente debido a su frecuente asociación con el síndrome metabólico. ^22^ En resumen, en la CMC es frecuente ver cardiomegalia con hipertrofia de VI y en muchos casos hipertrofia y dilatación de VD con agrandamiento de aurícula izquierda, alteraciones de las membranas celulares con disrupción de fibras miocárdicas y cicatrización con fibrosis parcial y deterioro de membranas subcelulares, diferente a las observadas en otras patologías.

### Fisiopatología

La CMC está caracterizada por una respuesta cardiaca alterada sobre todo frente a situaciones de estrés. La respuesta es el resultado de una combinación de factores:

-Disfunción autonómica

-Alteración de la composición de la membrana celular

-Defecto en los canales iónicos

-Sobreproducción de factores cardio-depresores

**Disfunción autonómica.** Una alteración frecuente es la activación del sistema renina-angiotensina-aldosterona (SRAA) y el incremento del tono simpático. El principal disparador sería el barorreceptor comprometido por la hipovolemia, con disminución de la presión arterial con una exposición prolongada del cardiomiocito a estrés hemodinámico que resulta en injuria miocárdica, desensibilización y down-regulation de los receptores beta adrenérgicos.

**Modificaciones de la membrana celular.**Hay una disminución de la fluidez y permeabilidad de la membrana celular secundaria al aumento del colesterol de membrana con disminución de la lecitina colesterol acil transferasa cuyo resultado es la

interferencia en la activación de varios receptores unidos a las membranas y a los canales iónicos. Los receptores muscarínicos también están afectados y son ellos los que controlan la actividad del nodo sino-auricular y la conduccion AV. La inflamación desencadena disfunción cardiaca en los cirróticos descompensados. Las hipótesis serían: alteración de la permeabilidad intestinal, el retardo en el reclutamiento de linfocitos en los ganglios mesentéricos y la traslocación bacteriana que llevan a una estimulación constante del sistema immune. Citoquinas y radicales libres de oxígeno pueden deteriorar la función miocárdica provocando una falla de la contractilidad.

### Defecto en los canales iónicos y cardiodepresores humorales

Los endocannabinoides (EC) están aumentados en los cirróticos asociados a la inflamación, ellos se unen al receptor CB1 reduciendo la función cardiaca sistólica y diastólica. El óxido nítrico (ON) está relacionado con la inflamación y el aumento de sus concentraciones produce una disminución en la respuesta del músculo papilar. Monóxido de carbono (CO), óxido nítrico (ON), endocannabinoides y la homeostasis alterada del calcio intracelular resultan en activación de la apoptosis celular.

En la CMC hay una circulacion hiperdinámica y el compromiso cardíaco se explica por dos anomalías principales en esta relación en red que tienen hígado y corazón: la insuficiencia hepática y la hipertensión portal. Debido a la primera hay insuficiente síntesis de proteínas y un metabolismo alterado de ciertas moléculas, proteínas y lípidos; debido a la segunda hay congestión intestinal. ^23^

La **insuficiencia hepática** produce aumento de los ácidos biliares y esto altera los miofilamentos cardíacos. El polipéptido cotransportador de sodio/taurocolato es un transportador de membrana que altera la circulación enterohepática de los ácidos biliares y promueve fibrosis hepática y a su vez, la elevación en plasma de ácidos biliares como el taurocolato y el ácido cólico reducen la liberación de calcio del retículo sarcoplásmico y el influjo de calcio tisular respectivamente, disminuyendo la contractilidad miocárdica. ^24-26^ Además, los ácidos biliares en exceso estimulan los receptores muscarínicos M2, lo que reduce la producción de AMPc intracelular y disminuyen la contractilidad cardíaca y pueden provocar arritmias. ^25^ Por otro lado, la disfunción contráctil ocurre por una inversión en el consumo metabólico de ácidos grasos y glucosa por el miocardio y los ácidos biliares en exceso alteran esta relación de la oxidación de sustratos: mayor oxidación de glucosa y menos de ácidos grasos (la inversa es lo normal). En las membranas de los cardiomiocitos, hay una alteración de la relación colesterol:fosfolípidos, lo que produce rigidez de la membrana y alteración del funcionamiento de los receptores β-adrenérgicos y disminución de la producción intracelular de AMPc. ^25-28^ La Titina, una gran proteína que interviene en la contracción-relajación de la célula miocárdica y es regulada por la proteína kinasa A (PKA), está disminuida en la CMC; también el colágeno tipo III, que es más complaciente que el rígido colágeno tipo I, está disminuido, lo que explicaría las alteraciones en la función diastólica en

la CMC. ^29-34^ Por otro lado, la cadena pesada Miosina también se ve alterada en la CMC: la isoforma α-Miosina que es más rápida y fuerte en la contracción miocárdica, es reemplazada por la más lenta y débil β-Miosina. ^33, 34^

**Figura 2:**
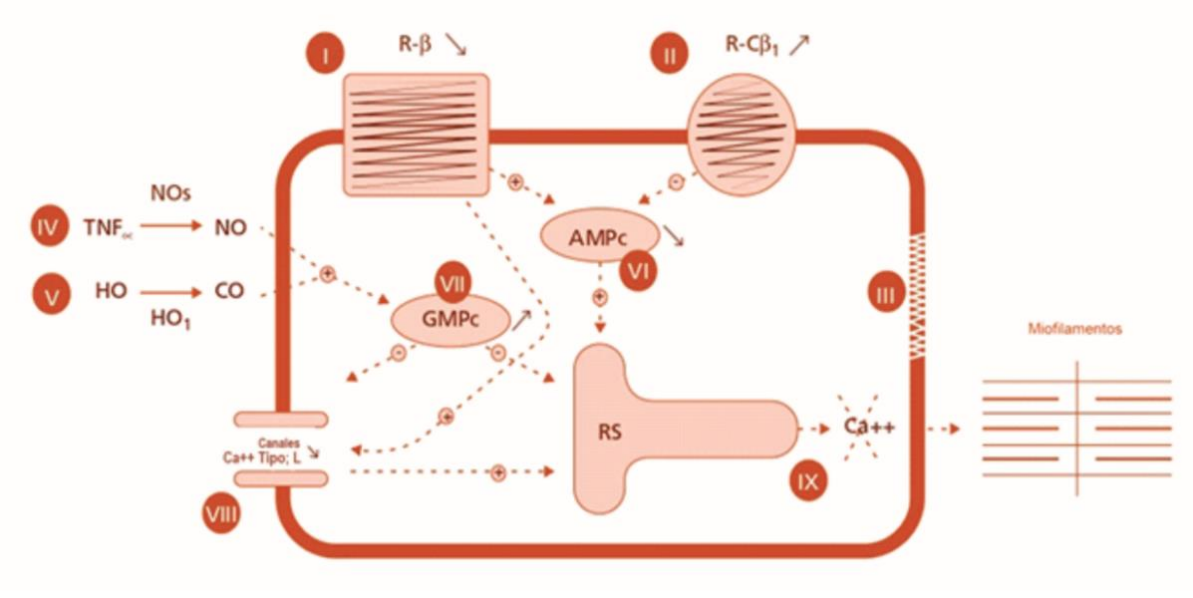
Principales mecanismos implicados en el defecto de contracción de los cardiomiocitos en presencia de Cardiomiopatía Cirrótica. I) Disminución y alteración de receptores beta. II) Sobreexpresión del sistema endocannabinoide con unión a receptores CB-1. III) Alteración de la membrana del miocito. IV y V) Aumento de los niveles de NO y CO. VI y VII) Aumento de la tasa de AMPc y reducción de la tasa de cGMP. VIII) Disminución de los canales de calcio dependientes de voltaje. IX) Todo lo que conduce a un defecto en la liberación de calcio sarcoplásmico hacia el citosol y por lo tanto, al vínculo entre la actina y la miosina. Receptores R-b: receptores beta-adrenérgicos. R-Cb1: receptores cannabinoides tipo I. TNFα: factor de necrosis tumoral alfa, NOs: óxido nítrico sintetasa. NO: óxido nítrico. HO: heme oxigenasa. HO1: heme oxigenasa 1. CO: monóxido de carbono. AMPc: adenosina monofosfato cíclico. cGMP: monofosfato de guanosina cíclico. RS: retículo sarcoplasmático. Modificada de Marchetta S, et al. Rev Med Lieja 2015; 70: 86-91, con autorización. ^43^

La **hipertensión portal** debido a la congestión intestinal y aumento de su permeabilidad con traslocación bacteriana y de endotoxinas y alteración del microbioma, produce un estímulo inflamatorio con depósitos de monocitos/macrófagos en el miocardio y la liberación de citoquinas inflamatorias como TNFα, IL-1β e IL-6, que asociados con un disbalance en favor de la apoptosis, reducen la contractilidad cardíaca. ^19, 31^ Además, hay una sobreproducción de óxido nítrico (ON) y éste, que a bajas concentraciones genera AMPc y contribuye a la apertura de los canales de calcio y mejora la contractilidad miocárdica, a altas concentraciones estimula la producción de GMPc reduciendo la sensibilidad de los miofilamentos al calcio e inhibe la contracción cardíaca debida a los receptores β-adrenérgicos. ^32, 33^ De manera similar, el monóxido de carbono (CO), producto de la heme oxigenasa (HO), activa a la guanilato ciclasa soluble y aumenta los niveles de GMPc con el mismo resultado. Por el contrario, NO, CO y cannabioides endógenos están aumentados en la circulación periférica, especialmente en la esplácnica, lo que contribuye a la vasodilatación periférica. Además, la galectina-3, una betagalactosidasa ligada a la leptina, se encuentra aumentada en los pacientes cirróticos y correlaciona clínicamente con la disfunción sistólica y diastólica. ^34, 35^ Los receptores β-adrenérgicos al ser activados por el sistema simpático estimulan a la adenyl ciclasa generando AMPc el que activa a la PKA y ésta fosforila a proteínas relacionadas con la contractilidad como la proteína C ligada a la miosina, troponina I, receptores de rianodina, canales de calcio tipo L y fosfolamban; en los corazones cirróticos el exceso en la estimulación crónica simpática provoca una disminución en la densidad de receptores β-adrenérgicos (down-regulation) y se han detectado anticuerpos anti receptores β1-adrenérgicos, lo que correlaciona con los niveles plasmáticos elevados de NT-proBNP, caída de la FEVI, y alteración de la relación E/A en el ecocardiograma en pacientes con CMC. ^36^ También los receptores cannabinoides están aumentados en los corazones de animales de experimentación y se observó que inhibiendo al CB1 que promueve inflamación y disfunción cardíaca, mejora la contractilidad. ^37^ El calcio extracelular ingresa al citoplasma a través de los canales de calcio tipo L y gatilla la liberación de calcio del retículo sarcoplásmico para iniciar la contracción cardíaca; en la cirrosis los canales de calcio tipo L están disminuidos en cantidad y alterados funcionalmente y también están alteradas las corrientes de potasio, lo que reduce la contractilidad en el primer caso y prolonga el intervalo QT en el segundo. ^25, 37-41^ Figura 2.

En resumen, la causa subyacente más importante es la congestión intestinal con incremento de la permeabilidad y la traslocación bacteriana que conduce a un fenotipo inflamatorio de cirrosis. Allí se producen mediadores inotrópicos negativos como citoquinas, NO, CO y EC. Una segunda oleada son los defectos en la síntesis de proteínas y lípidos con anomalías de la membrana plasmática y en la estructura y función intracelular del cardiomiocito, afectando los receptores beta adrenérgicos, canales iónicos, proteínas como titina, colágeno, cadenas pesadas de miosina, con una vía final anormal que incluye la cinética del calcio a través de la señalización del factor nuclear Kappa Beta.

## Clínica

La CMC es una entidad subclínica en gran parte de su evolución y la mayoría de los pacientes son diagnosticados durante un episodio de descompensación especialmente frente a una infección, trasplante hepático o TIPS. Muchos de los síntomas cardíacos no se diferencian de la enfermedad cirrótica subyacente y así los pacientes pueden permanecer aparentemente asintomáticos por mucho tiempo, aunque en realidad tienen síntomas cardíacos sutiles que no son adecuadamente valorados. Estos pacientes con cirrosis moderada o avanzada, en clase B o C de Child-Pugh, sin cardiopatía conocida previamente, presentan comúnmente intolerancia al esfuerzo con disnea y/o fatiga fácil o su empeoramiento, edema periférico e ingurgitación yugular en el contexto de la HTP. En etapas avanzadas, la ascitis con aumento de la presión intra-abdominal y la vasoconstricción afectan a los riñones y pueden desencadenar el SHR. La aparición de disfunción diastólica (DD) parece tener mal pronóstico, especialmente si se acompaña de hipoalbuminemia.^44,45^ En estadios más avanzados hay disfunción sistólica asociada a la DD y una marcada reducción del débito cardíaco con insuficiencia cardíaca.^44-48^

### Limitaciones

Esta revisión tiene algunas limitaciones. Primero, esta no es una revisión sistemática y algunos artículos relevantes pueden no haber sido citados y, en segundo lugar, no se realizó una evaluación formal de la calidad de la literatura.

## Discusión y/o Conclusión

El concepto de CMC fue descripto por Samuel S. Lee y desde entonces se realizaron numerosas investigaciones en animales de experimentación y en humanos. Su prevalencia no está clara, pero sería alrededor del 30-50% en pacientes con cirrosis avanzada. La anatomía patológica muestra alteraciones macroscópicas y microscópicas que son independientes de la etiología de la cirrosis, y la fisiopatología consiste en una circulación hiperdinámica con congestión intestinal, incremento de la permeabilidad y traslocación bacteriana con un fenotipo inflamatorio, alteraciones en los receptores de membrana de los cardiomiocitos y de las señales intracelulares que alteran la excitación-contracción miocárdica. Las manifestaciones clínicas cardíacas se superponen en parte con las de cirrosis por lo que es necesario tener en mente la sospecha para que el diagnóstico no sea tardío, sobre todo porque la descompensación cardíaca suele manifestarse en situaciones de estrés como cirugía, hemorragia, infecciones, trasplante hepático o colocación de TIPS. El Cirrhotic Cardiomyopathy Consortium definió los criterios para el diagnóstico y actualmente numerosas investigaciones están en curso para su refinamiento.

## References

[1] Tsao CW, Aday AW, Almarzooq ZI (2022). Heart Disease and Stroke Statistics-2022 Update: A Report From the American Heart Association. Circulation.

[2] Izzy M, VanWagner LB, Lin G, Altieri M, Findlay JY, Oh JK, Watt KD, Lee SS, Cirrhotic Cardiomyopathy Consortium (2020). Redefining Cirrhotic Cardiomyopathy for the Modern Era. Hepatology.

[3] Ruiz-del-Árbol L, Serradilla R (2015). Cirrhotic cardiomyopathy. World J Gastroenterol.

[4] Kowalski HJ, Abelmann WH (1953). The cardiac output at rest in Laennec's cirrhosis. J Clin Invest.

[5] Regan TJ, Levinson GE, Oldewurtel HA, Frank MJ, Weisse AB, Moschos CB (1969). Ventricular function in noncardiacs with alcoholic fatty liver: role of ethanol in the production of cardiomyopathy. J Clin Invest.

[6] Lee SS (1989). Cardiac abnormalities in liver cirrhosis. West J Med.

[7] Grose RD, Nolan J, Dillon JF, Errington M, Hannan WJ, Bouchier IA, Hayes PC (1995). Exercise-induced left ventricular dysfunction in alcoholic and non-alcoholic cirrhosis. J Hepatol.

[8] Finucci G, Desideri A, Sacerdoti D, Bolognesi M, Merkel C, Angeli P, Gatta A (1996). Left ventricular diastolic function in liver cirrhosis. Scand J Gastroenterol.

[9] Pozzi M, Carugo S, Boari G, Pecci V, de Ceglia S, Maggiolini S, Bolla GB, Roffi L, Failla M, Grassi G, Giannattasio C, Mancia G (1997). Evidence of functional and structural cardiac abnormalities in cirrhotic patients with and without ascites. Hepatology.

[10] Kaur H, Premkumar M (2022). Diagnosis and Management of Cirrhotic Cardiomyopathy. J Clin Exp Hepatol.

[11] Japp AG, Gulati A, Cook SA, Cowie MR, Prasad SK (2016). The Diagnosis and Evaluation of Dilated Cardiomyopathy. J Am Coll Cardiol.

[12] Chahal D, Liu H, Shamatutu C, Sidhu H, Lee SS, Marquez V (2021). Review article: comprehensive analysis of cirrhotic cardiomyopathy. Aliment Pharmacol Ther.

[13] Shahvaran SA, Menyhárt O, Csedrik L, Patai ÁV (2021). Diagnosis and Prevalence of Cirrhotic Cardiomyopathy: A Systematic Review and Meta-analysis. Curr Probl Cardiol.

[14] Cesari M, Frigo AC, Piano S, Angeli P (2021). Prevalence and prognostic value of cirrhotic cardiomyopathy as defined according to the proposed new classification. Clin Exp Hepatol.

[15] Razpotnik M, Bota S, Wimmer P, Hackl M, Lesnik G, Alber H, Peck-Radosavljevic M (2021). The prevalence of cirrhotic cardiomyopathy according to different diagnostic criteria. Liver Int.

[16] Ripoll C, Catalina MV, Yotti R, Olmedilla L, Pérez-Peña J, Lo Iacono O, Rincón D, García-Fernández MA, Bermejo J, Bañares R (2008). Cardiac dysfunction during liver transplantation: incidence and preoperative predictors. Transplantation.

[17] Merli M, Calicchia A, Ruffa A, Pellicori P, Riggio O, Giusto M, Gaudio C, Torromeo C (2013). Cardiac dysfunction in cirrhosis is not associated with the severity of liver disease. Eur J Intern Med.

[18] Caramelo C, Fernandez-Muñoz D, Santos JC, Blanchart A, Rodriguez-Puyol D, López-Novoa JM, Hernando L (1986). Effect of volume expansion on hemodynamics, capillary permeability and renal function in conscious, cirrhotic rats. Hepatology.

[19] Gaskari SA, Liu H, D'Mello C, Kunos G, Lee SS (2015). Blunted cardiac response to hemorrhage in cirrhotic rats is mediated by local macrophage-released endocannabinoids. J Hepatol.

[20] Ortiz-Olvera NX, Castellanos-Pallares G, Gómez-Jiménez LM, Cabrera-Muñoz ML, Méndez-Navarro J, Morán-Villota S, Dehesa-Violante M (2011). Anatomical cardiac alterations in liver cirrhosis: an autopsy study. Ann Hepatol.

[21] Lunseth JH, Olmstead EG, Abboud BBF (1958). A study of heart disease in one hundred eight hospitalized patients dying with portal cirrhosis. AMA Arch Intern Med.

[22] Wehmeyer MH, Heuer AJ, Benten D, Püschel K, Sydow K, Lohse AW, Lüth S (2015). High Rate of Cardiac Abnormalities in a Postmortem Analysis of Patients Suffering From Liver Cirrhosis. J Clin Gastroenterol.

[23] Wong F, Liu P, Lilly L, Bomzon A, Blendis L (1999). Role of cardiac structural and functional abnormalities in the pathogenesis of hyperdynamic circulation and renal sodium retention in cirrhosis. Clin Sci (Lond).

[24] Wong F, Girgrah N, Graba J, Allidina Y, Liu P, Blendis L (2001). The cardiac response to exercise in cirrhosis. Gut.

[25] Liu H, Nguyen HH, Yoon KT, Lee SS (2022). Pathogenic Mechanisms Underlying Cirrhotic Cardiomyopathy. Front Netw Physiol.

[26] Mokdad AA, Lopez AD, Shahraz S, Lozano R, Mokdad AH, Stanaway J, Murray CJ, Naghavi M (2014). Liver cirrhosis mortality in 187 countries between 1980 and 2010: a systematic analysis. BMC Med.

[27] Salhab A, Amer J, Lu Y, Safadi R (2022). Sodium+/taurocholate cotransporting polypeptide as target therapy for liver fibrosis. Gut.

[28] Zavecz JH, Battarbee HD (2010). The role of lipophilic bile acids in the development of cirrhotic cardiomyopathy. Cardiovasc Toxicol.

[29] Gorelik J, Harding SE, Shevchuk AI, Koralage D, Lab M, de Swiet M, Korchev Y, Williamson C (2002). Taurocholate induces changes in rat cardiomyocyte contraction and calcium dynamics. Clin Sci (Lond).

[30] Sheikh Abdul Kadir SH, Miragoli M, Abu-Hayyeh S, Moshkov AV, Xie Q, Keitel V, Nikolaev VO, Williamson C, Gorelik J (2010). Bile acid-induced arrhythmia is mediated by muscarinic M2 receptors in neonatal rat cardiomyocytes. PLoS One.

[31] Desai MS, Mathur B, Eblimit Z, Vasquez H, Taegtmeyer H, Karpen SJ, Penny DJ, Moore DD, Anakk S (2017). Bile acid excess induces cardiomyopathy and metabolic dysfunctions in the heart. Hepatology.

[32] Ma Z, Lee SS, Meddings JB (1997). Effects of altered cardiac membrane fluidity on beta-adrenergic receptor signalling in rats with cirrhotic cardiomyopathy. J Hepatol.

[33] Glenn TK, Honar H, Liu H, ter Keurs HE, Lee SS (2011). Role of cardiac myofilament proteins titin and collagen in the pathogenesis of diastolic dysfunction in cirrhotic rats. J Hepatol.

[34] Honar H, Liu H, Zhang ML, Glenn TK, Ter Keurs HEDJ, Lee SS (2020). Impaired myosin isoform shift and calcium transients contribute to cellular pathogenesis of rat cirrhotic cardiomyopathy. Liver Int.

[35] Huo S, Shi W, Ma H, Yan D, Luo P, Guo J, Li C, Lin J, Zhang C, Li S, Lv J, Lin L (2021). Alleviation of Inflammation and Oxidative Stress in Pressure Overload-Induced Cardiac Remodeling and Heart Failure via IL-6/STAT3 Inhibition by Raloxifene. Oxid Med Cell Longev.

[36] Huang X, Thansamay S, Yang K, Luo T, Chen S (2019). Measurement of Exhaled Nitric Oxide in Cirrhotic Patients with Esophageal and Gastric Varices. Biomed Res Int.

[37] Liu H, Ma Z, Lee SS (2000). Contribution of nitric oxide to the pathogenesis of cirrhotic cardiomyopathy in bile duct-ligated rats. Gastroenterology.

[38] Ansari U, Behnes M, Hoffmann J, Natale M, Fastner C, El-Battrawy I, Rusnak J, Kim SH, Lang S, Hoffmann U, Bertsch T, Borggrefe M, Akin I (2018). Galectin-3 Reflects the Echocardiographic Grades of Left Ventricular Diastolic Dysfunction. Ann Lab Med.

[39] Zivlas C, Triposkiadis F, Psarras S, Giamouzis G, Skoularigis I, Chryssanthopoulos S, Kapelouzou A, Ramcharitar S, Barnes E, Papasteriadis E, Cokkinos D (2017). Left atrial volume index in patients with heart failure and severely impaired left ventricular systolic function: the role of established echocardiographic parameters, circulating cystatin C and galectin-3. Ther Adv Cardiovasc Dis.

[40] Ma L, Liu X, Wu Q, Hu X, Liu H, Zhang J, Lee SS (2022). Role of Anti-Beta-1-Adrenergic Receptor Antibodies in Cardiac Dysfunction in Patients with Cirrhotic Cardiomyopathy. J Cardiovasc Transl Res.

[41] Ward CA, Ma Z, Lee SS, Giles WR (1997). Potassium currents in atrial and ventricular myocytes from a rat model of cirrhosis. Am J Physiol.

[42] Zardi EM, Abbate A, Zardi DM, Dobrina A, Margiotta D, Van Tassell BW, Afeltra A, Sanyal AJ (2010). Cirrhotic cardiomyopathy. J Am Coll Cardiol.

[43] Marchetta S, Altieri M, Weil-Verhoeven D, Mouhat B, Piérard L, Thévenot T (2021). La cardiomyopathie cirrhotique: de nouvelles approches diagnostiques. Hépato-Gastro et Oncologie Digestive.

[44] Karagiannakis DS, Papatheodoridis G, Vlachogiannakos J (2015). Recent advances in cirrhotic cardiomyopathy. Dig Dis Sci.

[45] Karagiannakis DS, Vlachogiannakos J, Anastasiadis G, Vafiadis-Zouboulis I, Ladas SD (2013). Frequency and severity of cirrhotic cardiomyopathy and its possible relationship with bacterial endotoxemia. Dig Dis Sci.

[46] Ruíz-del-Árbol L, Achécar L, Serradilla R, Rodríguez-Gandía MÁ, Rivero M, Garrido E, Natcher JJ (2013). Diastolic dysfunction is a predictor of poor outcomes in patients with cirrhosis, portal hypertension, and a normal creatinine.

[47] Yancy CW, Jessup M, Bozkurt B, Butler J, Casey DE Jr, Drazner MH, Fonarow GC, Geraci SA, Horwich T, Januzzi JL, Johnson MR, Kasper EK, Levy WC, Masoudi FA, McBride PE, McMurray JJ, Mitchell JE, Peterson PN, Riegel B, Sam F, Stevenson LW, Tang WH, Tsai EJ, Wilkoff BL, American College of Cardiology Foundation, American Heart Association Task Force on Practice Guidelines (2013 Oct 15). 2013 ACCF/AHA guideline for the management of heart failure: a report of the American College of Cardiology Foundation/American Heart Association Task Force on Practice Guidelines. J Am Coll Cardiol.

[48] Bozkurt B, Coats AJS, Tsutsui H, Abdelhamid CM, Adamopoulos S, Albert N, Anker SD, Atherton J, Böhm M, Butler J, Drazner MH, Michael Felker G, Filippatos G, Fiuzat M, Fonarow GC, Gomez-Mesa JE, Heidenreich P, Imamura T, Jankowska EA, Januzzi J, Khazanie P, Kinugawa K, Lam CSP, Matsue Y, Metra M, Ohtani T, Francesco Piepoli M, Ponikowski P, Rosano GMC, Sakata Y, Seferović P, Starling RC, Teerlink JR, Vardeny O, Yamamoto K, Yancy C, Zhang J, Zieroth S (2021). Universal definition and classification of heart failure: a report of the Heart Failure Society of America, Heart Failure Association of the European Society of Cardiology, Japanese Heart Failure Society and Writing Committee of the Universal Definition of Heart Failure: Endorsed by the Canadian Heart Failure Society, Heart Failure Association of India, Cardiac Society of Australia and New Zealand, and Chinese Heart Failure Association. Eur J Heart Fail.

